# In vitro antibiofilm and bacteriostatic activity of diacerein against *Enterococcus faecalis*

**DOI:** 10.1186/s13568-023-01594-z

**Published:** 2023-08-12

**Authors:** Chunyan Fu, Yuxi Xu, Hao Zheng, Xinyi Ling, Chengzhi Zheng, Leihao Tian, Xiaobin Gu, Jiabei Cai, Jing Yang, Yuanyuan Li, Peiyu Wang, Yuan Liu, Yongliang Lou, Meiqin Zheng

**Affiliations:** 1 Eye Hospital and School of Ophthalmology and Optometry, Wenzhou Medical University, Wenzhou, China; 2 National Clinical Research Center for Ocular Diseases, Eye Hospital, Wenzhou Medical University, Wenzhou, China; 3 Wenzhou Key Laboratory of Sanitary Microbiology, Key Laboratory of Laboratory Medicine, Ministry of Education, School of Laboratory Medicine and Life Sciences, Wenzhou Medical University, Wenzhou, China

**Keywords:** *Enterococcus faecalis*, Biofilm, Diacerein, RNA-seq, *Esp*, *LuxS*

## Abstract

*Enterococcus faecalis* is one of the main pathogens that causes hospital-acquired infections because it is intrinsically resistant to some antibiotics and often is capable of biofilm formation, which plays a critical role in resisting the external environment. Therefore, attacking biofilms is a potential therapeutic strategy for infections caused by *E. faecalis*. Current research indicates that diacerein used in the treatment of osteoarthritis showed antimicrobial activity on strains of gram-positive cocci in vitro. In this study, we tested the MICs of diacerein using the broth microdilution method, and successive susceptibility testing verified that *E. faecalis* is unlikely to develop resistance to diacerein. In addition, we obtained a strain of *E. faecalis* HE01 with strong biofilm-forming ability from an eye hospital environment and demonstrated that diacerein affected the biofilm development of HE01 in a dose-dependent manner. Then, we explored the mechanism by which diacerein inhibits biofilm formation through qRT-PCR, extracellular protein assays, hydrophobicity assays and transcriptomic analysis. The results showed that biofilm formation was inhibited at the initial adhesion stage by inhibition of the expression of the *esp* gene, synthesis of bacterial surface proteins and reduction in cell hydrophobicity. In addition, transcriptome analysis showed that diacerein not only inhibited bacterial growth by affecting the oxidative phosphorylation process and substance transport but also inhibited biofilm formation by affecting secondary metabolism, biosynthesis, the ribosome pathway and *luxS* expression. Thus, our findings provide compelling evidence for the substantial therapeutic potential of diacerein against *E. faecalis* biofilms.

**Supplementary Information:**

The online version contains supplementary material available at 10.1186/s13568-023-01594-z.

## Key Points

- Diacerein hinders the growth of *E. faecalis* and does not easily induce drug resistance.
- Diacerein inhibits biofilm formation of *E. faecalis* at the initial adhesion stage.
- Diacerein reduces the expression of the *esp* and *LuxS* genes and affects protein synthesis.

**Supplementary Information:**

The online version contains supplementary material available at 10.1186/s13568-023-01594-z.

## Introduction

*Enterococcus faecalis* widely colonizes the gastrointestinal system of humans and animals, but now it is one of the main pathogens causing nosocomial infection (Pöntinen et al. 2021). It is also infamous for its intrinsic resistance to some antibiotics, such as aminoglycosides and β-lactam-based antibiotics (Paganelli et al. 2012). Moreover, it is particularly worrying that antibiotic resistance rates of bacteria are increasing seriously, but not enough antibiotics are developed or put into clinical use (Willyard 2017).

Biofilms that make bacteria resistant to antibiotics or other harsh environmental conditions are specific structures made up of a community of organisms that consists of microcolonies and extracellular polymeric substances (EPS) (Yin et al. 2019; Karygianni et al. 2020). It has been reported that *E. faecalis* is often able to form biofilms, and biofilms have been identified as crucial virulence factors (Geraldes et al. 2022). Biofilms can stay on the surface of medical devices and are difficult to remove, so infections caused by *E. faecalis* are usually associated with the implantation of medical devices, including bacteraemia (Rosselli Del Turco et al. 2021), endocarditis (Fernández-Hidalgo et al. 2020), urinary tract infection (Fernández-Hidalgo et al. 2020), endodontic failure (Qi et al. 2021), postoperative intraocular infection (Lin et al. 2018), etc. Therefore, attacking the biofilm of *E. faecalis* is a key treatment strategy for enterococcal infections.

Anthraquinones have many biological activities, such as anticancer (Greco et al. 2021), antibacterial (Liu et al. 2015a, b; Li et al. 2021), antiviral (Li et al. 2014) and antibiofilm effects (Song et al. 2021). Diacerein, a derivative of anthraquinones, possesses a significant anti-inflammatory effect and is currently used for the treatment of osteoarthritis (Almezgagi et al. 2020). Recent research indicates that diacerein has antimicrobial activity against strains of gram-positive cocci in vitro (Zhang et al. 2019), which suggests that it has potential as an antibiotic. However, further experiments are needed to prove this hypothesis, and there is no corresponding study on the antibiofilm effect of diacerein. Due to the urgent need for new antibiotics to treat biofilm-mediated infections of *E. faecalis*, in the present study, we investigated the antibacterial and antibiofilm effects of diacerein and explored the mechanism of inhibiting the growth and biofilm formation of *E. faecalis* that was isolated from an eye hospital environment and had strong biofilm-forming ability.

## Materials and methods

### Bacterial strains and general reagents

*Staphylococcus epidermidis* ATCC 35984 (RP62A), *S. epidermidis* ATCC 12228 and six strains of *E. faecalis*, HE01 (CGMCC 1.60110), CYQ30, 36, 61, 142, and 162 which were isolated from an eye hospital Eye Hospital, were stored in the biobank of the Affiliated Eye Hospital of Wenzhou Medical University, Wenzhou, China. *E. faecalis* ATCC29212 was purchased from Wenzhou Kangtai Biotechnology Co., Ltd. (Wenzhou, China). All strains were identified by automatic microbial mass spectrometry (Autof ms600, Autobio, Zhengzhou, China).

Diacerein was purchased from push biotechnology Co., Ltd (Chengdu, China). Tryptic soy broth (TSB) and Mueller Hinton broth (MHB) were purchased from Solarbao Technology Co., Ltd (Beijing, China). The antibacterial reagents tetracycline, ampicillin, chloramphenicol, vancomycin, doxycycline, linezolid and levofloxacin were purchased from Wenzhou Kangtai Biotechnology Co., Ltd.

### Determining MICs of diacerein and other drugs

The minimum inhibitory concentration (MIC) of diacerein against seven strains of *E. faecalis* was determined by broth microdilution methods (Zhang, Liu et al. 2019). Briefly, each bacterial strain was subcultured using MHB, and 100 μl of the bacterial diluent (2 × 10^5^ CFU/ml) was added to the 96-well plate. The stock solution containing 20480 µg diacerein in 1 ml dimethyl sulfoxide (DMSO) was prepared, and serial twofold dilutions of the working solution were diluted with MHB. Then, 100 μl of a working solution of diacerein was added to the bacterial wells. The contrasted cells were treated with the corresponding concentration of DMSO.

The plates were incubated at 37 ℃ for 24 h. The MICs were defined as concentrations of drugs at which there was no visible growth. Meanwhile, a microplate reader (spectra max 190, molecular devices, USA) was used to monitor bacterial growth at 600 nm.

The MICs of other drugs were determined according to the reagent instructions.

### Time killing curves

*Enterococcus faecalis* ATCC29212 and HE01 (1.0 × 10^5^ CFU/ml) were incubated with DMSO, 1/2 MIC, MIC, 2 × MIC, and 4 × MIC of diacerein at 37 ℃ in 24-well plates, and at specified time points, the bacterial solution was continuously diluted with sterile PBS. Ten microlitres of diluted bacterial solution was spread on LB plates, and all dilutions had three replicates. Then, we placed plates at 37 ℃ for 24 h and counted the bacterial colonies.

### Induced resistance assay

We used increasing MIC values to reflect the resistance development of *E. faecalis* ATCC29212 and HE01 to diacerein (Liu et al. 2015a, b). The MIC values were observed after bacteria with different concentrations of diacerein were cocultured for 24 h. Meanwhile, bacteria cultured with sub-MIC diacerein were used for the detection of the MIC of the next generation. The process was repeated for 20 passages, and resistance was defined as an increase in the original MIC of more than fourfold (Koh et al. 2013; Li et al. 2021).

### Biofilm formation assay

Seven strains of overnight-cultured *E. faecalis* were diluted to 1 × 10^5^ CFU/ml using TSB supplemented with 0.5% glucose (0.5% TSBG) (Gélinas et al. 2021). Then, 200 μl of culture was added to a 96-well plate, and the plate was incubated for 24 h at 37 ℃. After incubation, the supernatants were removed from each well and washed twice with sterile PBS (Solarbao, Beijing, China). 200 µl of 25% formaldehyde were added to the wells to fix the biofilm for 10 min, and the formaldehyde was removed. Then, the cells were stained with 0.1% crystal violet for 15 min at room temperature and washed in running water. After the wells dried, the stained biofilms were resuspended in 200 μl of 33% glacial acetic acid–ethanol, and the biofilms were quantified using a microplate reader at OD570 (Liu et al. 2015a, b).

*Staphylococcus epidermidis* ATCC 35984 and ATCC 12228 were used as positive and negative controls for the biofilm formation assay (Chen et al. 2016). The strain with the strongest biofilm-forming ability was chosen for further study.

### Detection of the antibiofilm effects of diacerein

The anti-biofilm assay of diacerein was divided into two parts, including inhibiting biofilm formation and eradicating mature biofilms (Song et al. 2021). To measure biofilm formation inhibition, the method used to detect the effect of diacerein on biofilm formation for *E. faecalis* HE01 was the same as that used for the antibacterial activity assays, but the medium was changed to 0.5% TSBG. Then, the inhibition rate was calculated according to Fallah (Fallah et al. 2017). To measure the eradication of mature biofilms, 24 h mature biofilms were cultured with 0.5% TSBG containing different concentrations of diacerein or DMSO for 24 h at 37 ℃. After incubation, each well was stained with crystal violet, as described previously.

### Effect of diacerein on the growth cycle of biofilm development

This assay was based on that reported by Xiang (Xiang et al. 2017) with a few modifications. HE01 cells (1 × 10^5^ CFU/ml) were cultured in 96-well plates, and after a certain period of cultivation, each well was stained with crystal violet as described previously. The incubation time was 0 h, 2 h, 4 h, 8 h, 10 h, 12 h, 24 h, 36 h, 48 h, 60 h, 72 h, 84 h, 96 h, 108 h, or 120 h; there were 6 replicates for each time point.

The method used to detect the effect of diacerein on the growth cycle of biofilm formation was the same as above. However, after a certain period of cultivation, diacerein working solution at a test concentration of 32 μg/ml was added to the wells, and each well was incubated for 36 h. The timing of diacerein work solution addition was 0 h, 0.5 h, 1 h, 1.5 h, 2 h, 2.5 h, 3 h, 4 h, 5 h, 6 h, 8 h, 10 h, 12 h, or 24 h; there were 6 replicates for each time point. Wells without diacerein were used as controls.

### Bacterial surface hydrophobicity assay

The assay of bacterial surface hydrophobicity was performed as previously described (Mu et al. 2020), with a few modifications. HE01 cells (1.0 × 10^5^ CFU/ml) were incubated with diacerein working solution or the corresponding concentration of DMSO in a sterile test tube at 37 ℃ for 24 h without shaking.

Then, the bacterial solution was collected and centrifuged at 4000 rpm for 7 min at 4 ℃, the supernatant was removed, and the sample washed twice and resuspended in precooled sterile PBS. The turbidity was measured and designated H1. One millilitre of bacterial resuspension and 250 µl of n-hexadecane (Macklin, Shanghai, China) were added into glass tubes, shaken thoroughly on a vortex oscillator for 30 s, and allowed to sit for 10 min to layer. The turbidity of the underlying solution was measured and designated H2. The decrease in OD600 of the aqueous phase was used to reflect hydrophobicity (H%) as follows: H% = [(H1–H2)/H1] *100%. This experiment was performed with three independent repetitions.

### Identification of the main components of *E. faecalis* HE01 biofilms

To determine the main components of HE01 in 0.5% TSBG, biofilms were prepared in 96-well plates, treated with 200 µl of 10 mM sodium metaperiodate (Macklin, Shanghai, China), 10 U/ml DNase I (Beyotime, Shanghai, China), 10 μg/ml proteinase K (Beyotime, Shanghai, China), and 40 mM Tris–HCl (pH 8.0, Beyotime, Shanghai, China) as the control (Xiang et al. 2017); the plates were incubated at 37 ℃ for 1 h without shaking. Crystal violet staining was performed as previously described after incubation.

### Extracellular protein assay by fluorescence microscopy

HE01 cells (1.0 × 10^5^ CFU/ml) were treated with diacerein working solution for 24 h, and bacteria were collected at 8000 rpm for 7 min at 4 ℃, washed and resuspended in sterile PBS. Then, 5 μM Syto64 dye (Invitrogen, Shanghai, China) and 0.001% FITC dye (Invitrogen, Shanghai, China) were used to stain bacteria at 4 ℃ for 1 h (Xiang et al. 2017). Then, using PBS to remove excess dye, 10 μl of the stained bacterial solution was placed on a slide, dried and visualized by fluorescence microscopy.

### Transcriptomic sequencing and analysis

*Enterococcus faecalis* HE01 in 0.5% TSBG was inoculated with diacerein (32 µg/ml) or DMSO for 24 h at 37 ℃. Total RNA extracted with the RNAprep Pure Cell/Bacteria Kit (TianGen, Beijing, China) was delivered to Novogene Co., Ltd. (Beijing, China) for quality testing and transcriptome sequencing.

The resulting raw paired-end reads were trimmed and quality-filtered using fastp. Clean data were compared to the reference genome of *E. faecalis* V583 by Hista2. Then, rsubread was used to calculate gene expression in transcripts per kilobase million (TPM). Differential gene expression was analysed with DESeq2. Gene set enrichment analysis (GSEA) was performed with the Kyoto Encyclopedia of Genes and Genomes (KEGG) pathways as the annotation source. The differentially expressed genes (DEGs) with a log2-fold change (FC) > 2 and p. adjust < 0.05 were functionally categorized using Gene Ontology (GO) functional enrichment with eggNOG-mapper 2.1.9 as the annotation source.

### qRT-PCR

The reference genome (*E. faecalis* V583) lacked the enterococcal surface protein (*esp*) gene, but *esp* is critical in adhesion during biofilm formation (Ch’ng et al. 2019). Quantitative real-time PCR (qRT-PCR) was not only used to detect the expression of the *esp* gene after culture with diacerein but also to validate the RNA-seq data. Primer sequences are detailed in Additional file 1: Table S1. Total RNA was reverse-transcribed with a PrimerScript PT Reagent Kit with gDNA Eraser (TaKaRa, Dalian, China), and qPCR was performed in triplicate and quantified using TB Green (TaKaRa, Dalian, China) on an Applied Biosystems QuantStudio 6 Flex (ThermoFisher, USA). The PCR procedure was set up according to the manufacturer’s instructions, and relative quantitation was performed by the 2^−△△CT^ method. An internal control (EF_0629) was used to normalize the mRNA expression of selected genes.

### Statistical analysis

One-way analysis of variance in the current study was performed using GraphPad Prism 8.0.2, and statistical significance was determined by p < 0.05.

## Results

### Diacerein has antibacterial activity and does not easily induce resistance

**Fig. 1:**
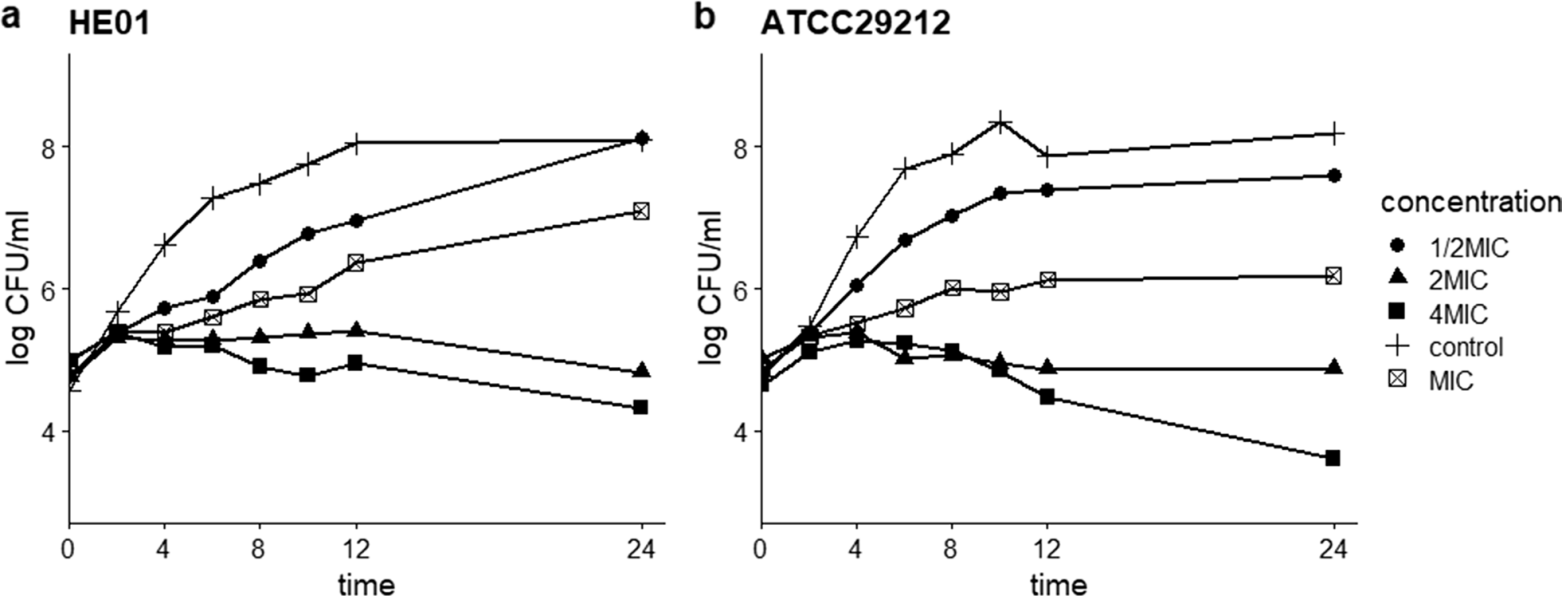
Time killing curves of *E. faecalis* strain HE01 (**a**) and ATCC29212 (**b**) treated with diacerein. The curves show changes in CFU/ml (Log) of bacteria at 0, 2, 4, 6, 8, 10, 12, and 24 h after culture with 0.5 × , 1 × , 2 × , and 4 × MIC diacerein. Bacteria treated with DMSO were included as a control

The antibacterial activity of diacerein in vitro was detected by determining its MIC against 7 strains of *E. faecalis*. The MIC values of diacerein were between 32 and 64 μg/ml (Additional file 1: Table S2). Time killing assays were conducted to evaluate the killing kinetics of diacerein against *E. faecalis* ATCC29212 and HE01. This curve showed that diacerein inhibited the growth of *E. faecalis* in a dose-dependent manner, and viable counts were continuously reduced by treatment with 2 × MIC and 4 × MIC diacerein (Fig. 1).

**Fig. 2:**
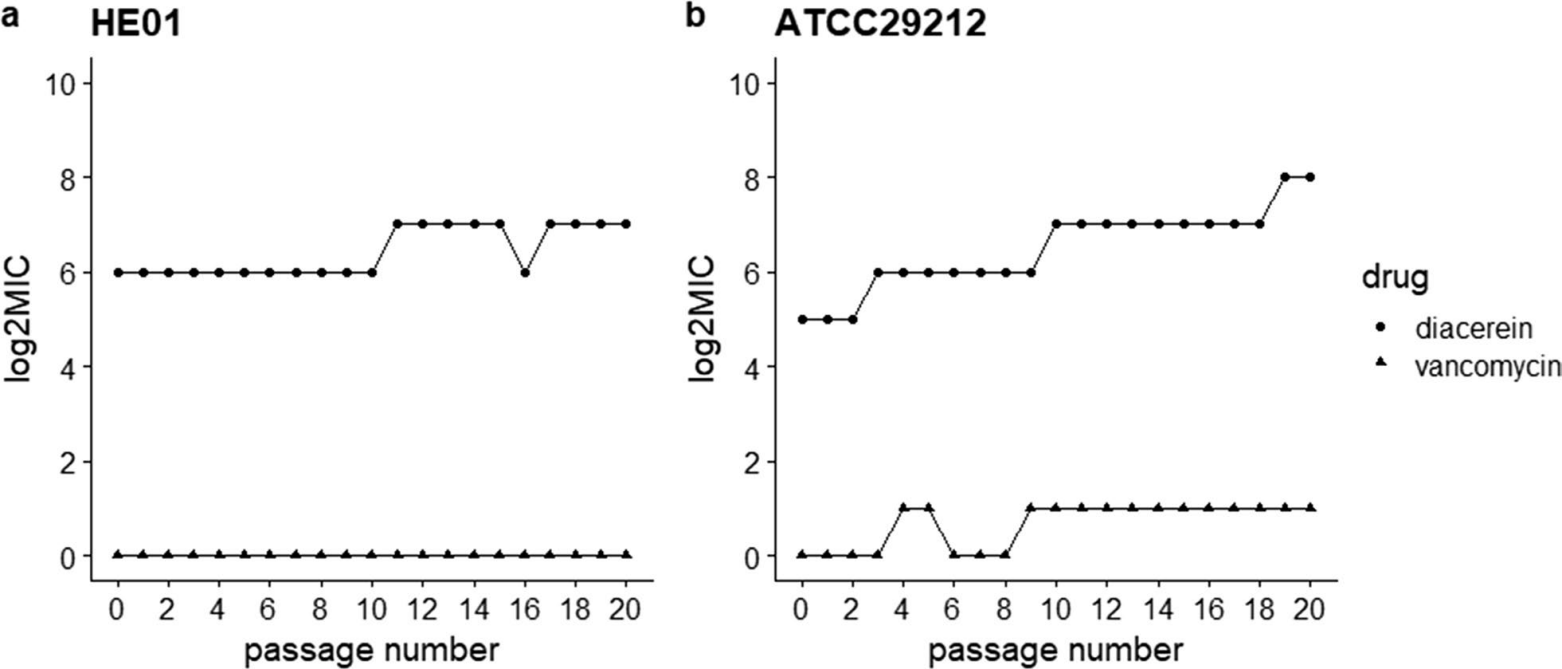
Curves show the continuous change in the MIC value of diacerein against *E. faecalis* HE01 and ATCC29212, which demonstrated multi-passage resistance selection of *E. faecalis* to diacerein. For HE01, no fourfold increase in MIC was observed. However, a fourfold increase in the MIC value for ATCC29212 was observed at passage 19

To investigate whether *E. faecalis* is susceptible to diacerein resistance, we performed a multi-passage resistance selection assay. For ATCC29212, at passage 19, the MIC showed a more than fourfold increase, which showed the emergence of resistance, but for HE01, resistance to diacerein was not induced within 20 passages. The above results indicated that *E. faecalis* did not easily generate resistance to diacerein (Fig. 2).

### Diacerein activity against *E. faecalis* HE01 biofilms

The biofilm forming capability of *E. faecalis* and the antibiofilm activity of diacerein were detected using crystal violet staining. Compared to the negative control, the results showed that all 7 strains had biofilm formation ability, and HE01 maintained the strongest biofilm-forming ability (Additional file 1: Fig. S1); thus, HE01 was used to verify the function of diacerein.

**Fig. 3:**
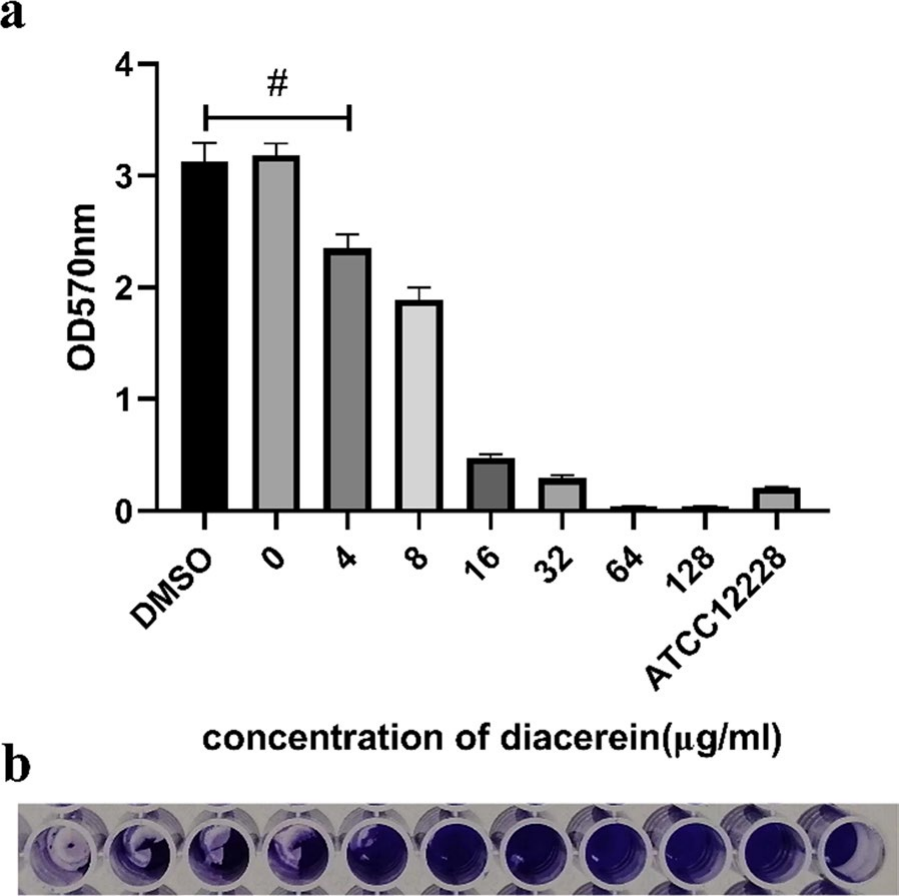
Diacerein inhibits HE01 biofilm formation and spreads mature biofilm. The biofilm inhibitory activity (**a**) and biofilm dispersive activity (**b**) of diacerein against HE01 were detected at serial concentrations. The measurement at OD570 nm was taken to measure the biofilm. The data represent the mean ± SD (n = 3), #p < 0.0001 (**a**). The concentration of diacerein used to eradicate mature biofilms was 1024 μg/ml to 4 μg/ml, from left to right, and for the remaining two wells, DMSO was added to one well, and no drug was added to the other (**b**)

Then, the antibiofilm activities of diacerein, including inhibiting biofilm formation and eradicating mature biofilms of HE01, were evaluated. Diacerein strongly inhibited the biofilm formation of HE01 at 0.5 × MIC (Fig. 3a). The inhibition rates were 97.13%, 91.04%, 43.40%, and 28.00% for 32, 16, 8 and 4 μg/ml, respectively. For preformed biofilms, diacerein induced dispersal. A concentration of diacerein greater than 32 μg/ml significantly dispersed the biofilm from the bottom of the 96-well plate (Fig. 3b).

### Diacerein inhibited biofilm development at the initial adhesion stage and at the early stage of microcolony formation

**Fig. 4:**
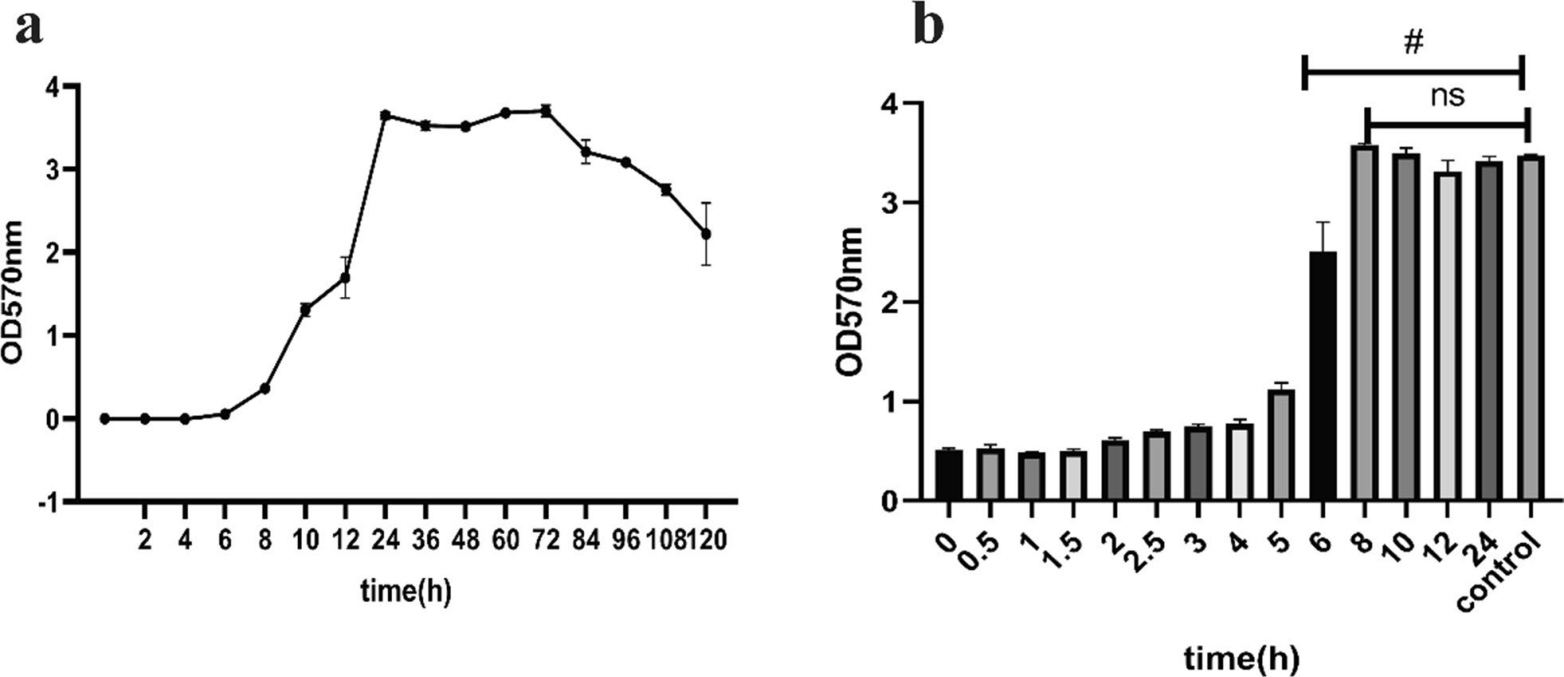
Diacerein affects the initial adhesion stage and early stage of microcolony formation during the process of HE01 biofilm development. The curve of biofilm growth shows the biofilm cycle of HE01 (**a**). The plot demonstrates the effect of diacerein on the growth of biofilm, and the time listed in this plot indicates the time at which the compound was added. The measurement at OD570 nm was taken to measure the biofilm. The data represent the mean ± SD (n = 6), #p < 0.0001(**b**)

To recognize the biofilm developmental stages of HE01 under culture conditions with 0.5% TSBG, we conducted assays as previously described (Xiang et al. 2017). Based on the results, similar to other bacteria, the biofilm formation process of HE01 can be divided into four stages (Ch’ng, Chong et al. 2019). The adhesion stage lasted from 0 to 4 h, the microcolony formation stage was between 4 and 12 h, the biofilm maturation stage was between 12 and 72 h, and the dispersal stage began after 72 h (Fig. 4a).

To detect the effect of diacerein on the growth cycle of biofilm formation, 32 μg/ml diacerein was added to cultivation at certain time periods. The addition of diacerein at 0 h–1.5 h resulted in the complete inhibition of biofilm formation. Diacerein reduced the biofilm gradually when added 2 h–6 h into biofilm development. After 8 h of incubation, the biofilm was resistant to diacerein (Fig. 4b). These results indicated that diacerein could inhibit biofilm development at the initial adhesion stage and at the early phase of microcolony formation.

### Diacerein decreases *esp* gene expression

**Fig. 5:**
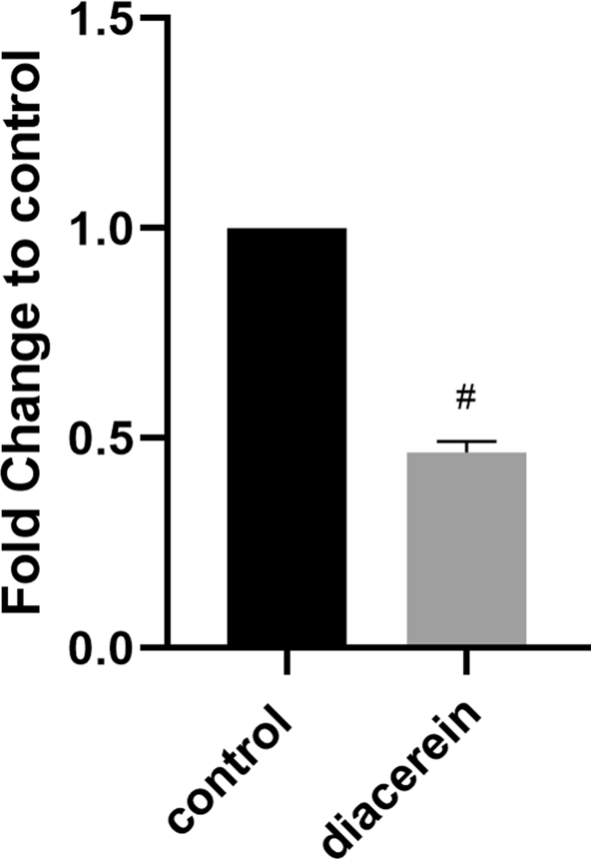
The expression of the *esp* gene was decreased in *E. faecalis* HE01 treated with diacerein. The data represent the mean ± SD (n = 3), # p < 0.001

First, we used PCR and agarose gel electrophoresis (AGE) to confirm the presence of the *esp* gene in the HE01 genome and in other strains isolated from the eye hospital of WMU (Additional file 1: Fig S2). Then, the expression of the *esp* gene of HE01 after treatment with diacerein was measured by qRT-PCR. The results showed that compared with the negative control, the expression of the *esp* gene was decreased (Fig. 5).

### Diacerein decreased the cell surface hydrophobicity of *E. faecalis* HE01 cells

**Fig. 6:**
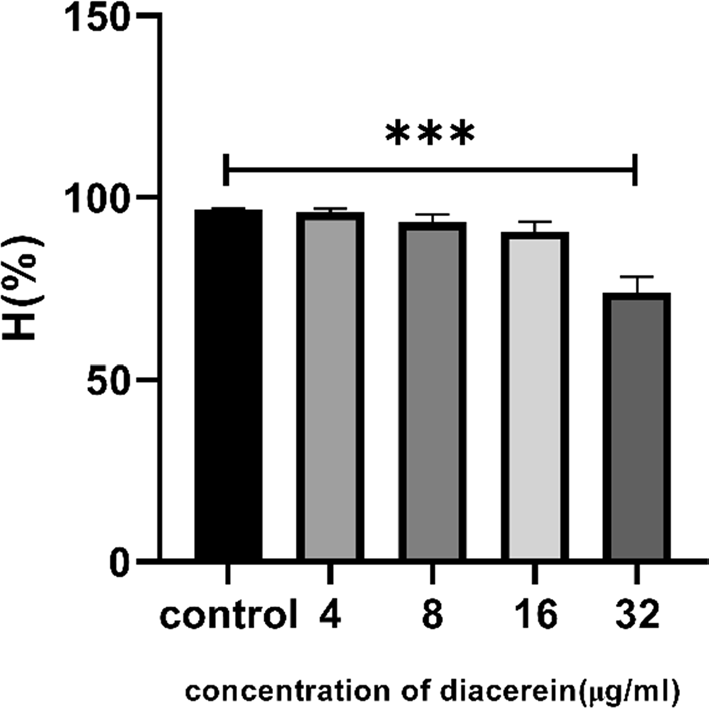
Diacerein decreased the surface hydrophobicity of HE01. The decrease in the absorbance at OD570 nm was taken as a measure of hydrophobicity. The data represent the mean ± SD (n = 3), *** p < 0.001

We measured the variation in HE01 surface hydrophobicity after culturing with diacerein using extraction of n-hexadecane. The results showed that HE01 cells treated with diacerein became less hydrophobic (Fig. 6), which partly explains why diacerein reduced biofilm formation.

### Protein is the main component of *E. faecalis* HE01 biofilm and diacerein reduces the production of extracellular proteins

**Fig. 7:**
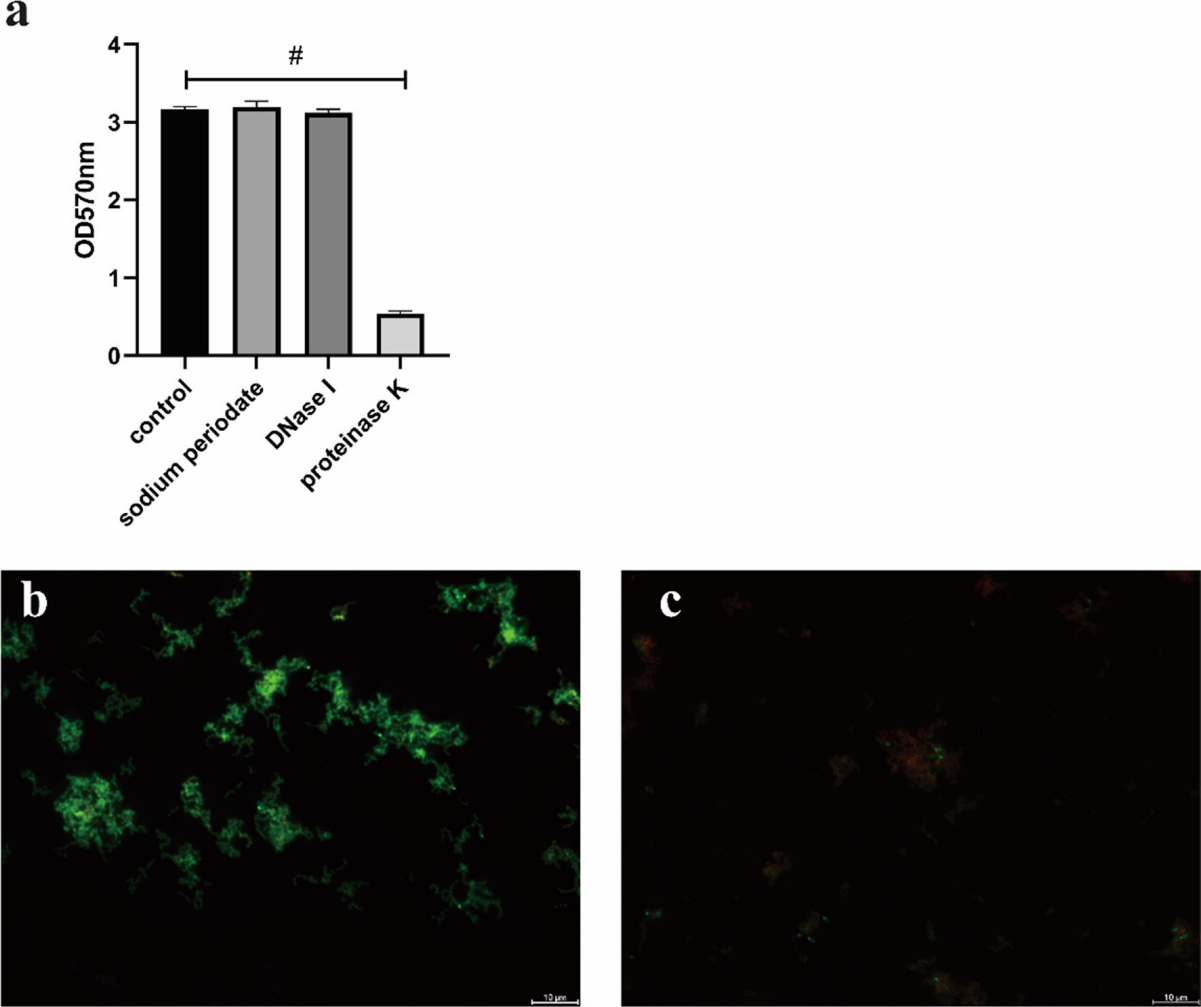
The main component of the extracellular matrix of the HE01 biofilm is protein, and diacerein decreases the production of extracellular proteins. After treating mature biofilms with proteinase K, DNase I, and sodium periodate, absorbance at OD570 nm was measured to evaluate residual biofilm (**a**). Extracellular proteins of stains ware detected by the change in green fluorescence, using intracellular DNA red fluorescence as internal reference. Compared with the control (**b**), the extracellular green fluorescence of *E. faecalis* was significantly reduced after treatment with diacerein (**c**)

Since the composition of the extracellular matrix of coccal biofilms varies under different culture conditions(Xiang et al. 2017), we used proteinase K, DNase I and sodium periodate to detect proteins, eDNA and polysaccharides, respectively, in the extracellular matrix of *E. faecalis* HE01 biofilms. According to the results, after treatment with proteinase K, the residual biofilm was minimal, and those treated with sodium periodate had almost no change (Fig. 7a).

After culture with diacerein or DMSO, we used Syto63 dye and FITC dye to stain intracellular DNA red and extracellular proteins green. Then, fluorescence microscopy was used to study the effect of diacerein on extracellular proteins. Green fluorescence surrounded the strain treated with DMSO, but it was diminished significantly when that strain was treated with diacerein (Fig. 7b, c), which indicates that diacerein reduces the production of extracellular proteins.

### Transcriptomic analysis

**Fig. 8:**
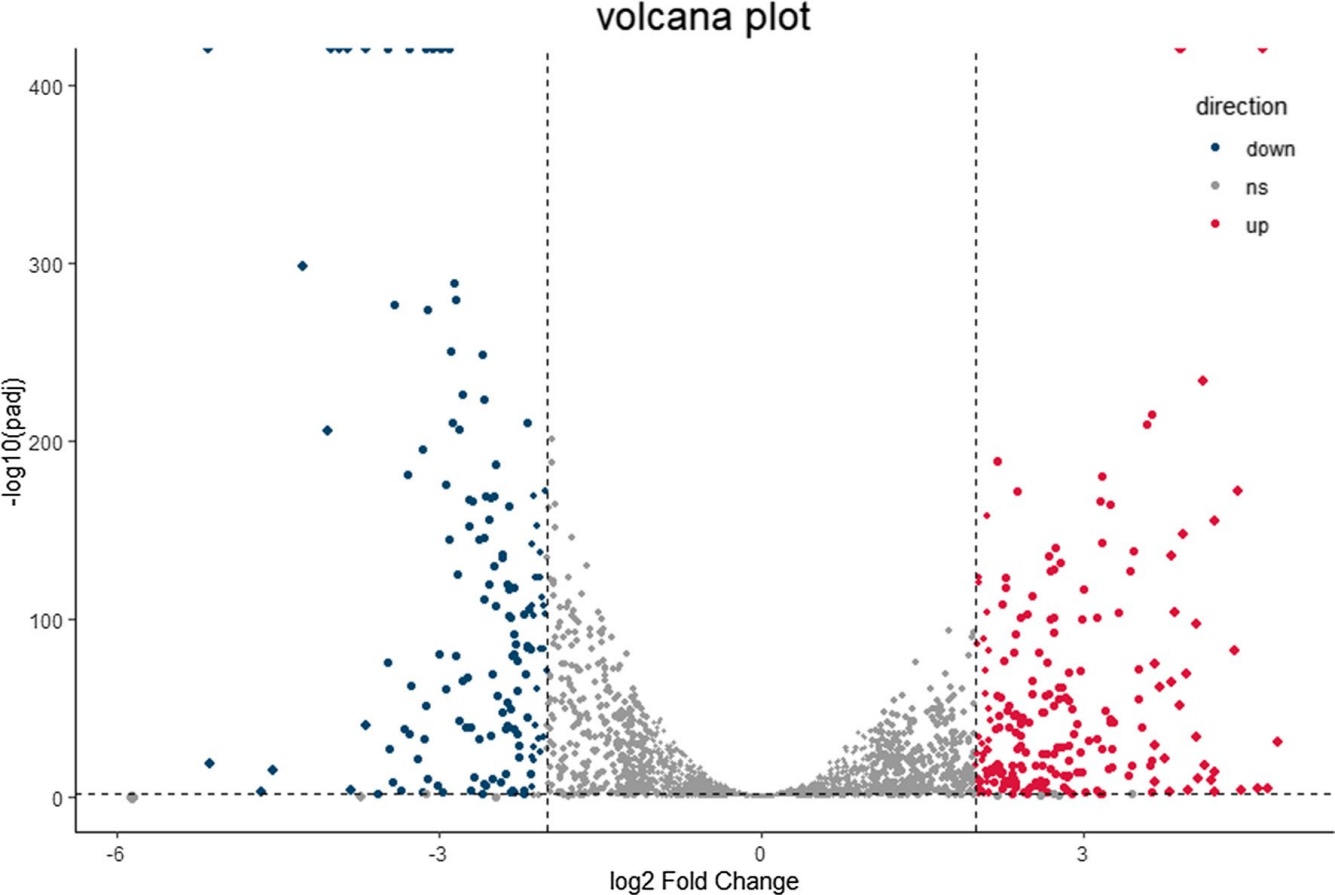
Volcano map showing the overall DEGs in HE01 with or without diacerein treatment. In this picture, the abscissa shows the log2-fold change in gene expression, and the ordinate shows the statistical test values. Each point represents a specific gene. Red dots, blue dots and grey dots indicate upregulated genes, downregulated genes and non-significantly changed genes, respectively. The farther the dot is located from the outside and top of the picture, the greater the significance of the difference in expression

To further study the mechanism by which diacerein inhibits *E. faecalis* biofilm formation, we analysed the gene expression of *E. faecalis* HE01 treated with or without diacerein (32 µg/ml) by RNA sequencing (RNA-seq). We first evaluated sequencing quality by the ratio of reads and reference genome sequences (Additional file 1: Table S3). The results showed that the ratio of each sample was greater than 85%, so the sequencing results were reliable. Transcript levels were normalized by calculating the TPM. Then, the expression levels of bacteria treated with diacerein were analysed relative to those treated with DMSO. A total of 2538 genes were analysed, and 363 genes were significantly affected by diacerein treatment (p. adjust < 0.05), including 217 upregulated genes and 146 downregulated genes, which are presented in a volcano map (Fig. 8). Transcriptomic analysis was consistent with the results of qRT-PCR (Additional file 1: Fig. S3).

### Enrichment analysis

**Fig. 9:**
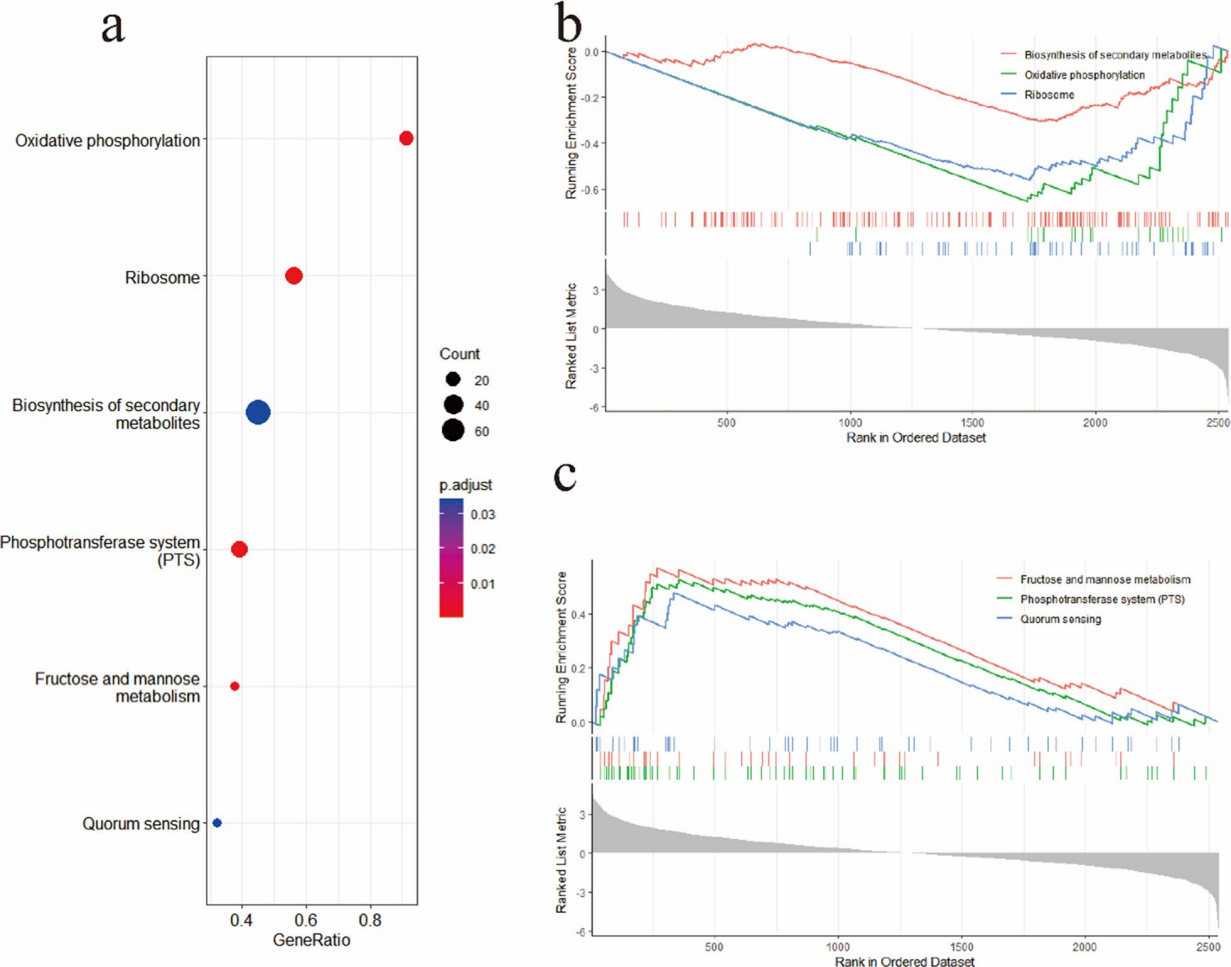
GSEA based on KEGG was used to detect changes in pathways. There were six significantly enriched pathways. The abscissa indicates the ratio of genes, while the ordinate indicates the GO function classification, and the sizes and colours of the dots indicate the number of enriched genes and p. adjust (**a**). The downregulated (**b**) and upregulated (**c**) pathways in the diacerein treatment group are shown

To determine which metabolic pathways of *E. faecalis* were affected by diacerein, we performed GSEA on the results of differential gene expression using KEGG pathways as the annotation source. Six pathways were significantly enriched (p. adjust < 0.05) (Fig. 9a), including three downregulated pathways (biosynthesis of secondary metabolites: efa01110, ribosome: efa03010 and oxidative phosphorylation: efa00190) (Fig. 9b) and three upregulated pathways (phosphotransferase system (PTS): efa02060, fructose and mannose metabolism: efa00051 and quorum sensing: efa02024) (Fig. 9c). The biosynthesis of secondary metabolites pathway, which is associated with metabolism and the biosynthesis of amino acids, glycolysis/gluconeogenesis, and the citrate (TCA) cycle, was the most enriched, which suggests that diacerein significantly affects this pathway to inhibit biofilm formation. The PTS pathway, which involves the phosphorylation of monosaccharides, was the second most significantly enriched pathway, followed by the ribosome pathway. Therefore, diacerein also blocked the ribosomal protein synthesis of *E. faecalis*, which constitutes the site of bacterial protein synthesis.

**Fig. 10:**
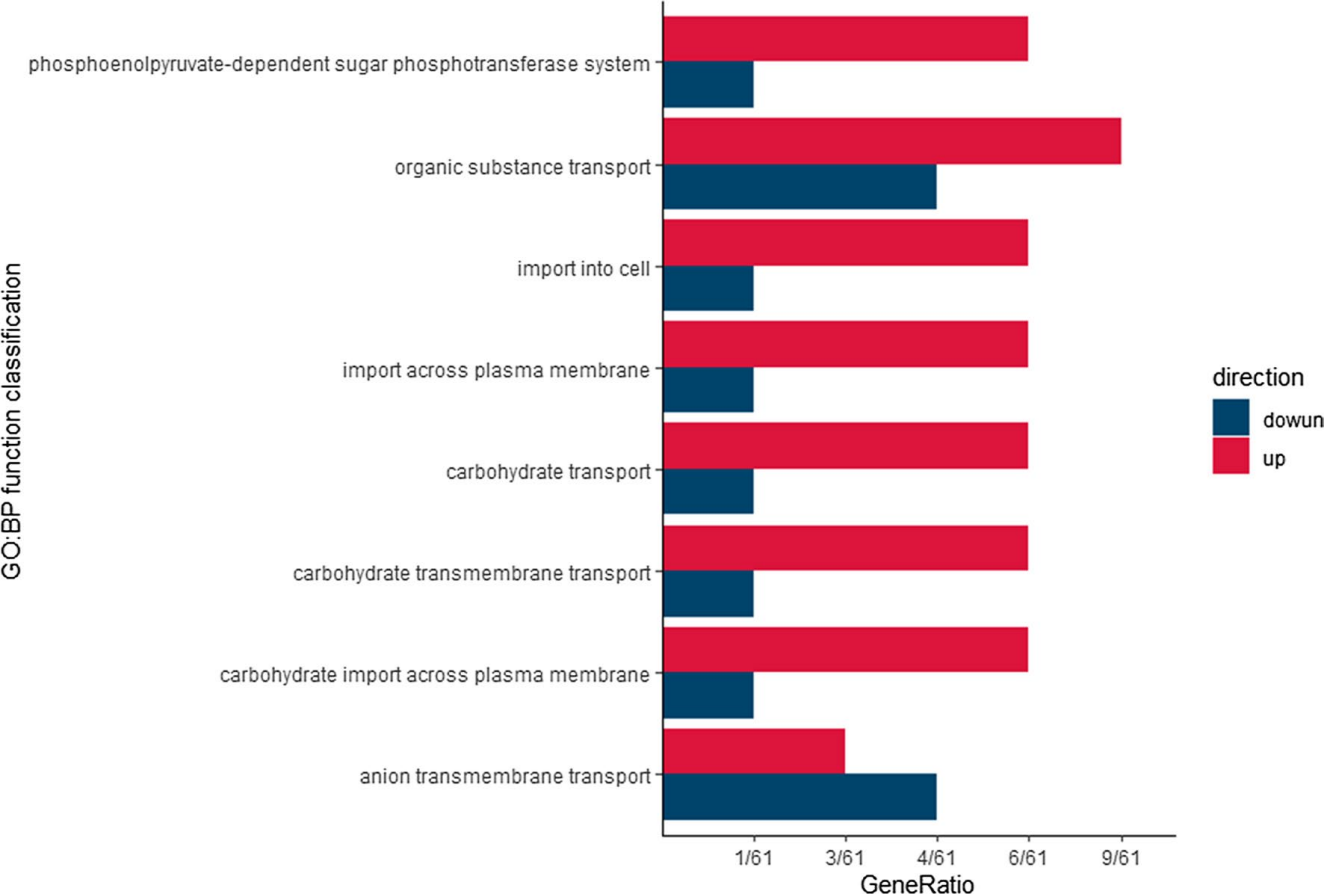
GO: BP enrichment analysis of DEGs. The abscissa indicates the gene ratio, while the ordinate indicates the GO function classification. Red and blue were used to mark up- and downregulation, respectively

GO functional enrichment analysis was used to detect the function of up- or downregulated DEGs (Gélinas et al. 2021), and when p-values were < 0.05, the GO function was considered significantly enriched (Li et al. 2021). A total of 363 genes were assigned to 8 GO terms, and all terms were biological processes (BP) (Fig. 10), including PTS and process of transportation. The process of organic substance transport was the most enriched, and 4 out of 13 term-related genes were downregulated, while 9 out of 13 genes were upregulated.

## Discussion

Due to the biofilm formation capability and increasing drug resistance by year to year of *E. faecalis *(Willyard 2017), new antibiotics that do not easily induce resistance and have antibiofilm activity are urgently needed for clinical treatment. Diacerein is an anthraquinone compound that has antibiofilm bacteriostatic effects on cocci (Song et al. 2021), so the antibiofilm ability of diacerein warrants further study.

Before exploring the antibiofilm ability, we preliminarily investigated the antibacterial activity of diacerein. The time-killing curve suggested that diacerein had rapid and dose-dependent bacteriostatic activity against *E. faecalis*, and the results of multi-passage resistance assays indicated that diacerein did not easily induce drug resistance within 10 successive passages. The MIC values of diacerein against *E. faecalis* ranged from 32 to 64 μg/ml, unrelated to other drugs, which suggests that its bacteriostatic mechanism was different from that of other drugs. The MBC of *E. faecalis* was more than 4 × MIC, suggesting that diacerein could not be judged as a bactericidal drug (Li et al. 2021); however, diacerein significantly inhibited bacterial growth. Transcriptomic analytic results indicated that diacerein inhibited bacterial growth by affecting material transport and energy metabolism; these conclusions were based on the results showing that the expression of the oxidative phosphorylation pathway was significantly decreased and the GO: BP function was significantly enriched in transmembrane transport after treatment with diacerein. However, the mechanism of diacerein against *E. faecalis* growth still needs more experimental verification.

To explore the antibiofilm activity of diacerein, we selected *E. faecalis* HE01, which has the strongest biofilm-forming ability, as the experimental strain. Biofilm formation could be significantly inhibited at sub-MIC levels, which indicates that the inhibitory effect of diacerein on biofilm formation was not dependent on bacteriostasis (Song et al. 2021), and we found that when diacerein was added at the initial adhesion stage or at the early phase of microcolony formation, the biofilm growth process was terminated.

Adhesion is the first step in biofilm formation, and several proteins have a vital role, such as endocarditis and biofilm-associated pilus (ebp) ABC, aggregation substance (Agg), SagA-like protein B (salB) and esp(Ch’ng, Chong et al. 2019, Geraldes, Tavares et al. 2022). Esp, as a virulence factor related to colonization, is prevalent in *E. faecalis *(Aung et al. 2023) and can increase the surface hydrophobicity of *E. faecalis *(Toledo-Arana et al. 2001). We confirmed by qRT-PCR that diacerein significantly reduced *esp* gene expression, which further indicated that diacerein could affect the formation of *E. faecalis* biofilms during the initial adhesion stage.

The EPSs of biofilms have three main components as follows: extracellular DNA (eDNA), proteins, and exopolysaccharides (Ch’ng, Chong et al. 2019). We confirmed by enzymolysis assay that the EPS of the HE01 biofilm was mainly composed of proteins under 0.5% TSBG culture conditions. Subsequently, we determined by fluorescence microscopy that diacerein inhibits extracellular protein production, leading to inhibition of biofilm formation. Moreover, transcriptomic analytic results indicated that the expression of the ribosome pathway, which is essential for protein synthesis, was significantly decreased in *E. faecalis* after treatment with diacerein. Diacerein decreased cell surface hydrophobicity, facilitating initial adhesion to hydrophobic surfaces and playing a vital role in biofilm formation in coccus (Mu et al. 2020); this change in hydrophobicity may also be related to the decreased expression of surface proteins because bacteria need certain proteins to maintain cell surface hydrophobicity, such as esp.

The quorum sensing system allowing bacteria to communicate through autoinducers (AIs), which can regulate the microbial community to synchronize transcription and other behaviours, is crucial to biofilm formation. The LuxS system is a quorum-sensing system of *E. faecalis*, and the LuxS enzyme is a key enzyme that catalyses the production of AI-2, which is used for bacterial communication (Ali et al. 2017). However, some genes involved in ATP synthesis, translation, wall/membrane biogenesis, and metabolism are also regulated by the LuxS enzyme. (Shao et al. 2012). In addition, it has been suggested that depletion of the *luxS* gene decreased the biofilm formation capability of *E. faecalis *(Yang et al. 2018). We demonstrated that diacerein significantly reduced luxS gene expression through qRT-PCR and transcriptomic analysis, despite upregulation of the quorum sensing pathway, which suggests its role in the inhibition of biofilm formation.

Proteomic studies have shown that using planktonic cells as a contrast, biofilm cells have higher expression levels of associated proteins, including those related to glycolysis, amino acid biosynthesis and metabolism, biosynthesis of secondary metabolites, and luxS-mediated quorum sensing (Suryaletha et al. 2019). In this study, the GSEA results indicated that the above biological processes, which were highly enriched in DEGs of biofilm cells, were decreased in *E. faecalis* treated with diacerein. This result indicated that diacerein decreases the proteins associated with these biological processes to inhibit biofilm formation.

In conclusion, we confirmed the bacteriostatic activity of diacerein and are the first to report that it inhibits the biofilm formation of *E. faecalis* during the initial adhesion stage by reducing the expression of the *esp* and *luxS* genes, which affects protein synthesis and multiple metabolic pathways in vitro. In addition, we observed that diacerein induced the shedding of mature biofilms of *E. faecalis*. We suggest that diacerein is a potential antibiotic against biofilms associated with *E. faecalis*, but the antibiofilm inhibition ability of diacerein in vivo and its ability to cause the mature biofilm to detach from material needs further verification.

### Supplementary Information

**Additional file 1: Figure S1.** The absorbance at 570 nm was used to judge the biofilm formation ability of 7 strains of *Enterococcus faecalis*. The higher the value, the stronger the biofilm formation ability, and vice versa. **Figure S2.** The assay of AGE confirmed the presence of *esp *gene in strains isolated from hospital, from left to right; marker, HE01, ATCC29212, CYQ30, CYQ36, CYQ61,CYQ142,CYQ162. **Figure S3.** The results of RNA-Seq were verified by qRT-PCR analysis. The genes for verification were selected from a list of genes with significant changes in transcription. Results were expressed as mean ± standard deviation of the sample in three replicates. **Table S1.** Primers used in this study. **Table S2.** MICs (μg/ml) of diacerein and other antibiotics to *E. faecalis. *Table S3. Ratio of sequencing data to reference genome.

## Data Availability

The raw data sets of transcriptome sequencing in this study have been uploaded to the NCBI database under the accession number PRJNA934180.
