# Supplementary material for: In vitro antibiofilm and bacteriostatic activity of diacerein against Enterococcus faecalis
Source: AMB Express. 2023 Aug 12;13:85. doi: 10.1186/s13568-023-01594-z (PMC10423188; doi:10.1186/s13568-023-01594-z)
Supplement: Supplementary file 1 — Additional file 1: Figure S1. The absorbance at 570 nm was used to judge the biofilm formation ability of 7 strains of Enterococcus faecalis. The higher the value, the stronger the biofilm formation ability, and vice versa. Figure S2. The assay of AGE confirmed the presence of esp gene in strains isolated from hospital, from left to right; marker, HE01, ATCC29212, CYQ30, CYQ36, CYQ61,CYQ142,CYQ162. Figure S3. The results of RNA-Seq were verified by qRT-PCR analysis. The genes for verification were selected from a list of genes with significant changes in transcription. Results were expressed as mean ± standard deviation of the sample in three replicates. Table S1. Primers used in this study. Table S2. MICs (μg/ml) of diacerein and other antibiotics to E. faecalis. Table S3. Ratio of sequencing data to reference genome. [file 13568_2023_1594_MOESM1_ESM.pdf]

AMB Express

In vitro antibiofilm and bacteriostatic activity of diacerein against *Enterococcus faecalis*

Chunyan Fu[1,2], Yuxi Xu[1,2], Hao Zheng[3], Xinyi Ling[3], Chengzhi Zheng[1,2], Leihao Tian[1,2], Xiaobin Gu[3], Jiabei Cai[1,2], Jing Yang[1,2], Yuanyuan Li[3], Peiyu Wang[1,2], Yuan Liu[1,2], Yongliang Lou[3] and Meiqin Zheng[1,2,3*]

[1]Eye Hospital and School of Ophthalmology and Optometry, Wenzhou Medical University, Wenzhou, China
[2]National Clinical Research Center for Ocular Diseases, Eye Hospital, Wenzhou Medical University, Wenzhou, China
[3]Wenzhou Key Laboratory of Sanitary Microbiology, Key Laboratory of Laboratory Medicine, Ministry of Education, School of Laboratory Medicine and Life Sciences, Wenzhou Medical University, Wenzhou, China

Correspondence:
Meiqin Zheng
e-mail: zmqlyllh@126.com
telephone: 86-13806694662

Table S1 Primers used in this study

gene		Primers sequence (5' to 3')
EF_1182	F	ACAAAATGGTGATGCGTTAGTCGA
	R	TCACGGATTTCTTGTGGTGAATGT
EF_1498	F	GATTCCTTGTCGGATAAAGACCGT
	R	ACTGTTCCCGCCATTAACTCAGA
EF_1500	F	GTGAATTCAAACAGCGAGCTTGATC
	R	TAGACGTGTAATTTCGCCACGTTC
EF_1492	F	ATCAAGGCAGCGGAAGATGC
	R	TGTTCAGTGCGCTTTGTTTCATCTA
EF_0696	F	CGATGCTTCGACACCAGGAC
	R	TGGAATTTCTTCGGCTAAACACTCTGA
EF_0485	F	GTGTTAGGAGCCGTAGGATTAGC
	R	CTTTGGTCGGTTGAGAAGTAGTGTC
esp	F	CAACCCTAAAGCAATAACTGGTG
	R	GCCCTTCTTCGGATCAACT
EF_0629	F	ATACAGTAACATTAAACAACGGCACTA
	R	CGTTCCAAACTTTCGAGGTAACAAA

Table S2 MICs (μg/mL) of diacerein and other antibiotics to *E. faecalis*

	diacerein		TCY	AMP	CHL	VAN	DOX	LNZ	LVX
	MIC	MBC	MIC	MIC	MIC	MIC	MIC	MIC	MIC
HE01	64	> 1024	8	0.5	32	1	≤0.25	4	> 4
ATCC29212	32	> 1024	16	0.5	4	2	4	2	0.5
CYQ30	64	> 1024	≤0.25	0.5	4	1	≤0.25	1	0.5
CYQ36	64	> 1024	32	0.5	4	1	4	1	0.5
CYQ61	64	> 1024	32	0.5	4	1	4	1	0.5
CYQ142	64	> 1024	32	0.5	2	1	4	1	0.5
CYQ162	32	> 1024	32	0.5	4	1	4	1	0.5

TCY, tetracycline; AMP, ampicillin; CHL, chloramphenicol; VAN, vancomycin; doxycycline, DOX; LNZ, linezolid; LVX, levofloxacin

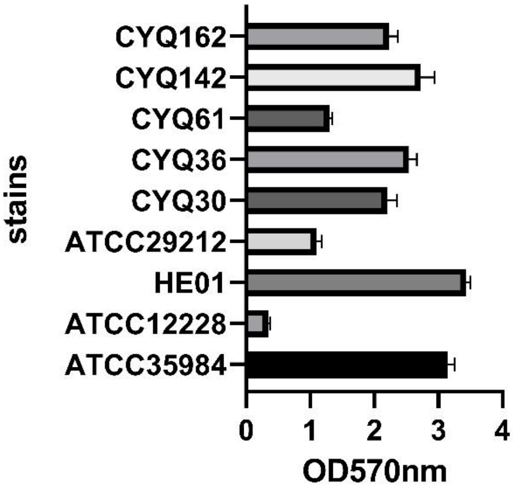

Fig.S1 The absorbance at 570nm was used to judge the biofilm formation ability of 7 strains of *Enterococcus faecalis*. The higher the value, the stronger the biofilm formation ability, and vice versa.

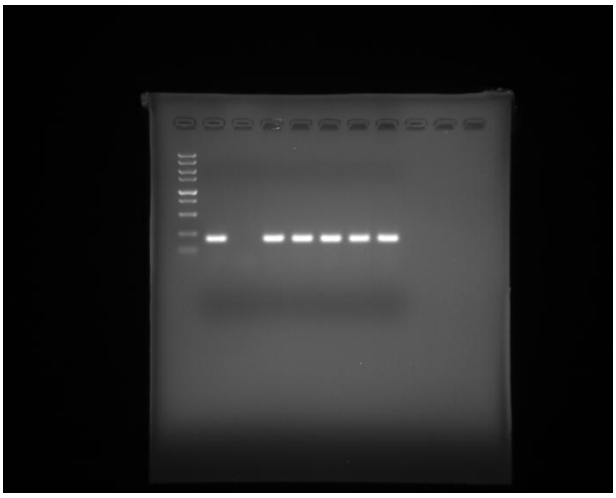

Fig. S2 The assay of AGE confirmed the presence of *esp* gene in strains isolated from hospital, from left to right; marker, HE01, ATCC29212, CYQ30, CYQ36, CYQ61,CYQ142,CYQ162.

Table S3 Ratio of sequencing data to reference genome

sample name	total pairs	mapped pairs	uniquely mapped pairs	total mapped pairs	mapped unpaired pairs	uniquely mapped pairs	total mapped rate(%)
control							
control_1	4424827	4157582	4117680	534490	100758	99639	95.1
control_2	5835740	5488554	5421628	694372	134460	132522	95.2
control_3	3942685	3702935	3621977	479500	89931	88203	95.06
diacerein							
diacerein_1	3567364	2971671	2944374	1191386	157301	154931	85.51
diacerein_2	4258845	3551132	3520676	1415426	187463	184805	85.58
diacerein_3	4275235	3606202	3577133	1338066	170452	168142	86.34

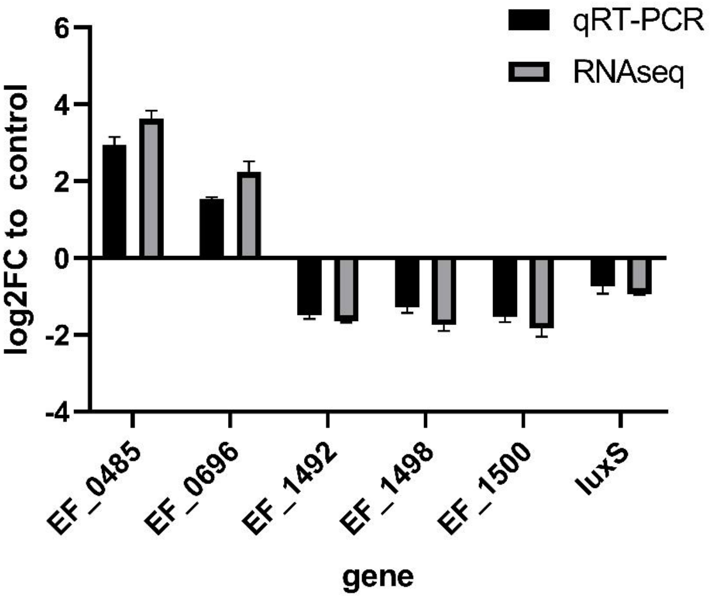

Fig.S3 The results of RNA-Seq were verified by qRT-PCR analysis. The genes for verification were selected from a list of genes with significant changes in transcription. Results were expressed as mean ± standard deviation of the sample in three replicates.
